# Case series: Pleural effusion caused by urinary ultrafiltrate in two cats without evidence of urinary obstruction, trauma, or simultaneous perinephric pseudocysts

**DOI:** 10.3389/fvets.2022.1038278

**Published:** 2022-11-21

**Authors:** Maureen A. Griffin, Michele A. Steffey, Kathryn L. Phillips, Philipp D. Mayhew, Kevin D. Woolard, Ann Della Maggiore

**Affiliations:** ^1^UC Davis Veterinary Medical Teaching Hospital, School of Veterinary Medicine, University of California-Davis, Davis, CA, United States; ^2^Department of Surgical and Radiological Sciences, School of Veterinary Medicine, University of California-Davis, Davis, CA, United States; ^3^Department of Pathology, Microbiology, and Immunology, School of Veterinary Medicine, University of California-Davis, Davis, CA, United States; ^4^MarQueen Emergency and Specialty Hospital, Roseville, CA, United States

**Keywords:** urothorax, hydrothorax, perinephric pseudocyst, Tc-DTPA, feline

## Abstract

**Objectives:**

To describe the diagnostic techniques, surgical treatments, and outcomes of two cats with recurrent pleural transudate caused by urinary ultrafiltrate.

**Animals:**

Two cats without evidence of trauma, urinary tract obstruction, or concurrent perinephric pseudocysts that were evaluated and treated for recurrent pleural transudate caused by urinary ultrafiltrate.

**Study design:**

Short case series.

**Methods:**

Multiphase contrast CT scan revealed leakage of contrast media from the kidneys bilaterally into the retroperitoneal spaces in both cats. Renal scintigraphy performed in one cat revealed progressive accumulation of ^99m^Tc diethylenetriamine penta-acetic acid (Tc-DTPA) in the pleural space. Exploratory laparotomy localized the leakage of fluid to renal capsular defects bilaterally in both cats. The retroperitoneum was incised bilaterally to promote fluid drainage into the peritoneum, and nephropexies were performed.

**Results:**

One cat had long-term survival with recurrent, though decreasing volumes of, pleural effusion. The second cat was euthanized 16 days postoperatively for progressive renal disease.

**Conclusion:**

The diagnosis of spontaneous urinary ultrafiltrate accumulation in the pleural space of cats without evidence of trauma, urinary tract obstruction, or concurrent perinephric pseudocysts has not previously been reported. The surgical correction described reduced but did not completely eliminate the rate of pleural effusion accumulation.

## Introduction

Transdiaphragmatic migration of fluid in dogs was described as early as 1929, but case reports of translocation of fluid from the urinary tract into the pleural space are very rare in dogs and cats (1, 2). There has been one feline case report of hemiurothorax associated with diaphragmatic hernia and renal prolapse, and two reports of urothorax in dogs with presumed traumatic etiologies (1, 3, 4). Several case reports of urothorax exist in people, and distinct etiologies have been proposed: obstructive urothorax, traumatic urothorax, and urothorax secondary to urinoma (5, 6). To date, no cases of hydrothorax secondary to urinary ultrafiltrate have been reported in people.

Spontaneous urothorax or urinary ultrafiltrate accumulation in the pleural space of cats without evidence of trauma, urinary tract obstruction, or concurrent urinoma or perinephric pseudocysts has not been reported. The objective of this report is to describe the diagnostic techniques, surgical treatments, and outcomes of two cats with recurrent pleural transudate caused by urinary ultrafiltrate.

## Case descriptions

### Cat 1

A 9-year-old male castrated domestic shorthair cat was presented to its local veterinarian for coughing and waxing and waning peripheral edema. Thoracic radiographs revealed pleural effusion. After three thoracocentesis events that each recovered between 300 and 420 cm^3^ of fluid, the cat was referred to the study institution.

At presentation the cat weighed 4.4 kg and had a body condition score of 4/9. A moderate amount of peripheral edema was present in its inguinal region. The cat exhibited increased respiratory effort, and decreased breath sounds were appreciated ventrally. A grade II/VI left parasternal systolic heart murmur was ausculted. Pleural effusion was detected bilaterally *via* thoracic ultrasound, and bilateral thoracocentesis drained 362 cm^3^ of translucent fluid from the pleural space. The fluid was classified as a pure transudate (protein 3 kg/m^3^, total nucleated cell count 90/mm^3^) and contained a creatinine of 29 g/m^3^, glucose of 1.32 kg/m^3^, and potassium of 2.7 mol/m^3^. Complete blood count showed a moderate normocytic, normochromic, nonregenerative anemia (hematocrit 22.8%; RR: 30–50%). Serum biochemistry panel revealed azotemia [consistent with IRIS stage 2 chronic kidney disease (CKD)] characterized by a creatinine of 28 g/m^3^ (RR: 12–22 g/m^3^) and BUN of 540 g/m^3^ (RR: 180–330 g/m^3^) (7). Urinalysis revealed a specific gravity of 1.031 with proteinuria (750 g/m^3^) and glucosuria (10,000 g/m^3^). Lactate dehydrogenase (LDH) evaluation was as follows: < 0.01 IU/cm^3^ in the effusion, 0.092 IU/cm^3^ in the urine, and 0.188 IU/cm^3^ in the blood. A NT-proBNP test (IDEXX, Westbrook, ME) was normal. Abdominal ultrasound showed bilaterally decreased renal corticomedullary distinction with cortical cysts and scant peritoneal effusion. Echocardiography revealed a mild myocardial irregularity and an impaired relaxation pattern of left ventricular diastolic filling (possibly age related or early heart disease).

**Figure 1:**
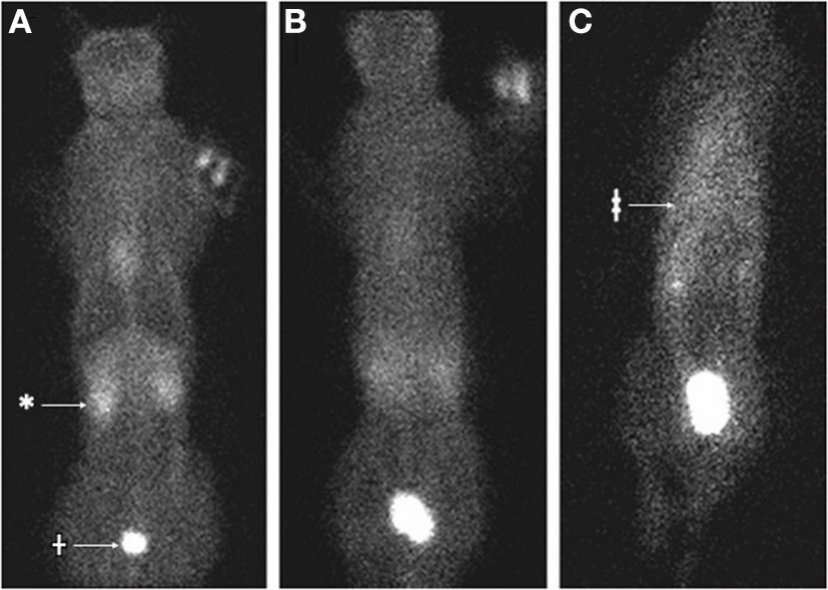
Nuclear scintigraphy of cat 1 following IV injection of ^99m^Tc diethylenetriamine penta-acetic acid (Tc-DTPA). **(A)** At 5 min following injection, the radiopharmaceutical is distributed through the body with increased signal in the kidneys (*) and urinary bladder (†). **(B)** At 30 min following injection, the distribution is homogenous without the signal void in the region of the lungs. **(C)** At 3 h following injection, there is more radiopharmaceutical accumulated in a triangular shape within the thoracic cavity (‡) compared to other tissues.

**Figure 2:**
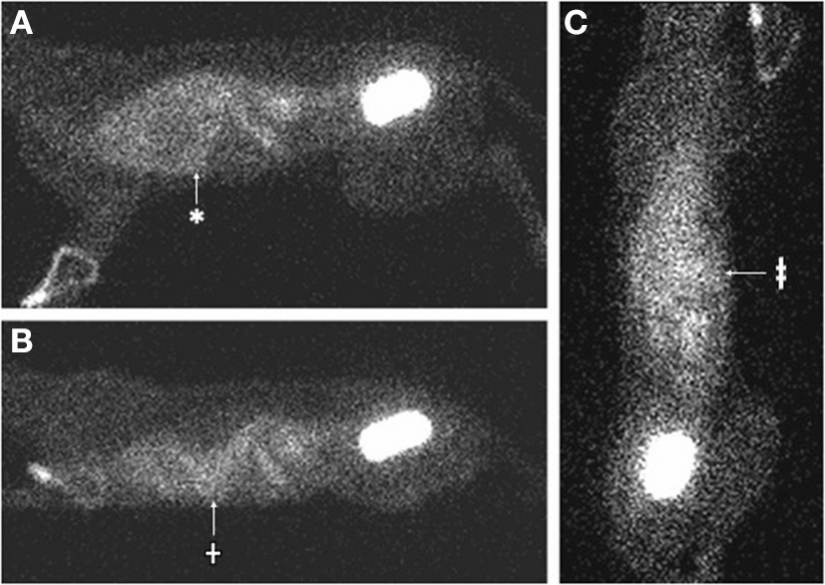
Nuclear scintigraphy images (cat 1) 4 h following IV injection of Tc-DTPA, demonstrating accumulation of mobile radiopharmaceutical within the pleural space. **(A)** The patient is in right lateral recumbency. There is increased radiopharmaceutical in the thoracic cavity (*), which faintly outlines the cardiac silhouette. **(B)** The patient is placed in sternal recumbency with the gamma camera horizontal to the patient. The radiopharmaceutical in the thorax moves with gravity to a dependent (ventral) position (†). **(C)** The patient is placed in dorsal recumbency on the gamma camera. The radiopharmaceutical in the thorax becomes more homogenously distributed and is evident in both hemithoraces (‡).

Renal scintigraphy was performed using ^99m^Tc diethylenetriamine penta-acetic acid (Tc-DTPA) to investigate for presence of urothorax. In this case, 2.25 millicuries Tc-DTPA were administered intravenously and 60 s static images were acquired using a low-energy, all-purpose collimator at 5 min, 20 min, and hourly until 4 h following injection (Figures 1, 2). Initially, there was blushing of the renal silhouettes as the Tc-DTPA accumulated within the kidneys with immediate extension of radiopharmaceutical in the urinary bladder. Over the next 4 h, technetium progressively accumulated within the pleural space, demonstrating a communication between the urinary tract and pleural space.

**Figure 3:**
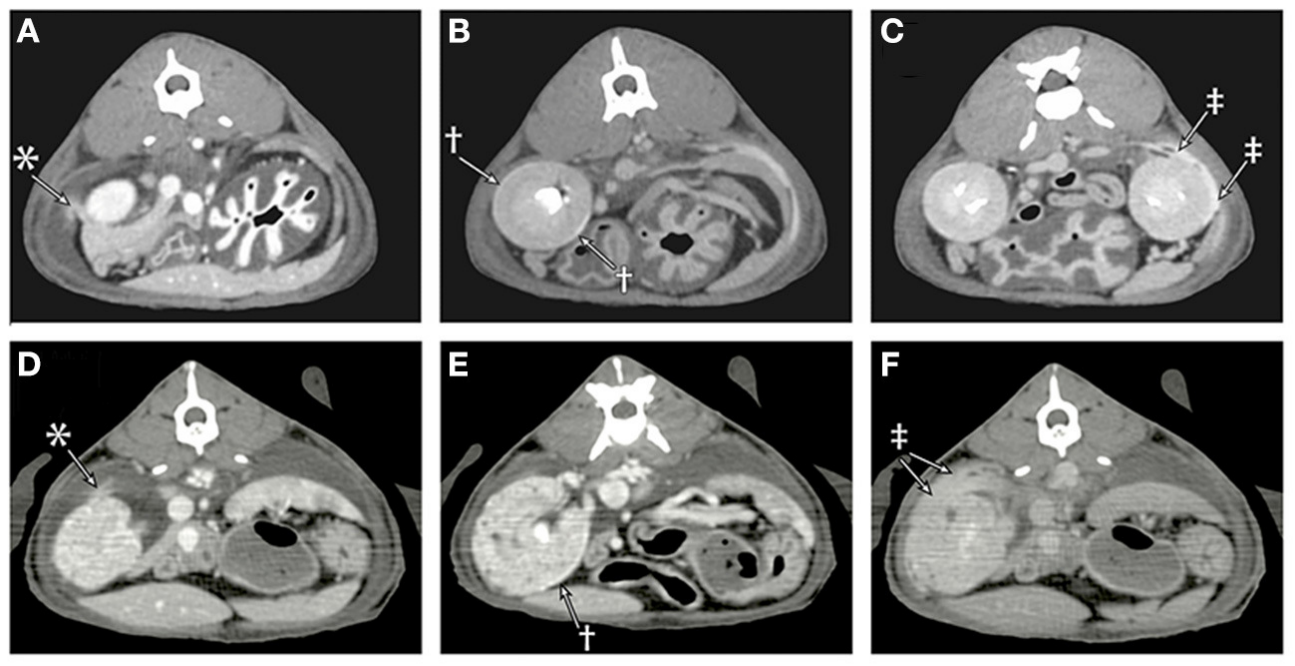
Sequential post-contrast CT images of cat 1 **(A–C)** and cat 2 **(D–F)** are provided during the venous phase **(A,D)**, 4 min **(B)**, 7 min **(E)**, and 10 min **(C,F)** following contrast administration. **(A)** In the venous phase images, there is a faint blush of contrast media (*) extending from the cranial pole of the right kidney into the adjacent retroperitoneal effusion. **(B)** A rim of contrast media (†) can be seen accumulating in the subcapsular space. **(C)** Contrast (‡) can be seen leaking from the patient's left kidney and accumulating in the retroperitoneal effusion. **(D)** Leakage of contrast (*) from the cranial pole of the right kidney into the retroperitoneal space is identified. **(E)** Degenerative renal changes are evident with numerous cortical cysts, and there is a thin rim of subcapsular contrast accumulation (†). **(F)** By the later phases, the retroperitoneal space is filled with contrast (‡).

Multiphase contrast-enhanced CT of the thorax and abdomen was performed with multiple post-contrast sequences (Figures 3A–C). On venous phase images, both kidneys had a thin outline of subcapsular contrast media with mild extravasation of the contrast into the retroperitoneal space. On delayed phase images, there was progressive accumulation of contrast media in the retroperitoneal effusion as well as an increase in Hounsfield units (from 8 to 16) of the pleural effusion. The pleura was mildly thickened, consistent with pleuritis from chronic pleural effusion, and fluid could be seen on either side of the plica vena cava. There were multiple small contrast filling defects within the renal cortical tissue consistent with renal cortical cysts, and no ureteral abnormalities were detected. Subcutaneous edema was noted in the pelvic limbs and perineal region.

**Figure 4:**
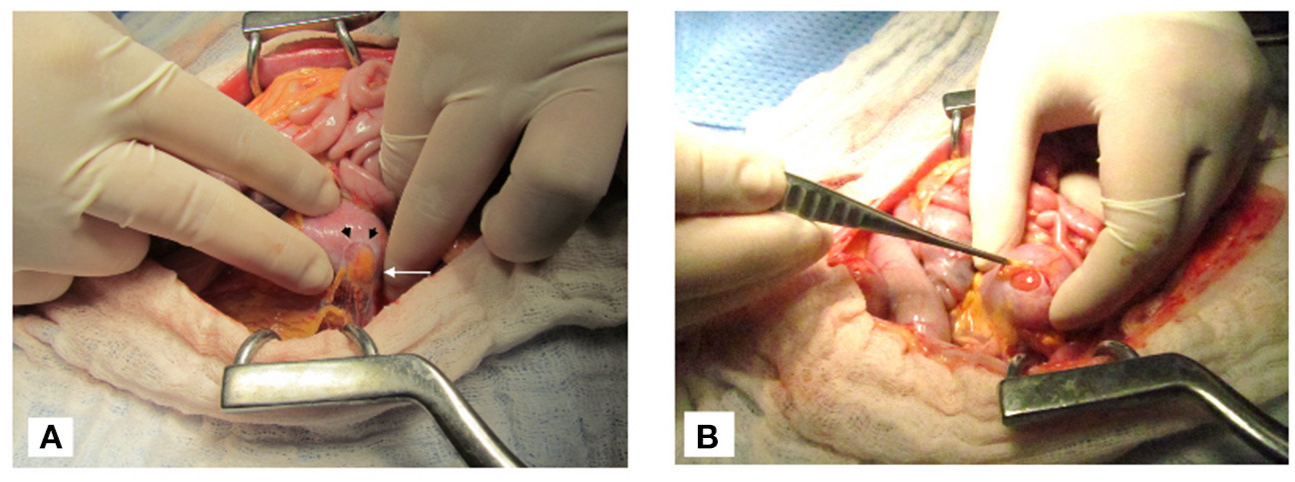
Intraoperative images of cat 1. **(A)** The edge of a renal capsular defect (black arrowheads) in the left kidney can be seen with adherence of retroperitoneal fat to the exposed renal parenchyma (white arrow). **(B)** After removal of adhesions, the renal parenchyma may be seen through the capsular defect.

Exploratory celiotomy revealed minimal translucent peritoneal effusion. No visible or palpable defects were identified in the substance of the diaphragm or at the caval foramen, esophageal hiatus, or aortic hiatus. The retroperitoneum appeared gelatinous bilaterally. After retroperitoneal incision, leakage of fluid was traced to circular defects in the cranial aspect of the renal capsules bilaterally. Retroperitoneal fat was adhered to the exposed renal parenchyma (Figure 4) and local retroperitoneal fibrosis was detected in these regions. Fluid was observed to be leaking from these defects and gentle probing revealed a lack of connection of the renal capsule surrounding the defects to the underlying renal parenchyma. Ultrafiltrate fluid was observed accumulating in this space before leaking into the retroperitoneal space. No ureteral or bladder leaks were identified.

No reconstructive options were available, so the retroperitoneum was incised from the level of each kidney to the diaphragm to encourage drainage of fluid from the retroperitoneum into the peritoneum and thereby minimize tracking into the pleural space. Due to the subsequent instability of the renal attachments to the body wall, bilateral nephropexies were performed between the retroperitoneal sublumbar musculature and caudal pole of each kidney using 3-0 polypropylene in a cruciate pattern. The abdomen was closed in routine fashion. A biopsy of the right renal capsule adjacent to the capsular defect revealed minimal chronic lymphocytic and plasmacytic perinephritis. The cat received a packed red blood cell transfusion during the procedure and recovered from anesthesia without complications.

The cat returned 11 days postoperatively for a recheck examination. No clinical signs of pleural effusion were exhibited, though a brief ultrasound scan revealed a moderate amount of pleural effusion and pockets of free peritoneal fluid. The cat presented again 16 days later with an increased respiratory effort and respiratory rate of 40 breaths per minute. A brief ultrasound exam revealed a moderate amount of pleural effusion and a scant amount of peritoneal effusion. Thoracocentesis was performed and 294 cm^3^ of clear fluid was removed.

The cat continued to produce small amounts of pleural effusion, and intermittent peripheral edema was reported during periods of increased pleural effusion. The owner elected to place a pleural drainage catheter and port system ~2 months after the initial surgery. Pleural effusion was managed for 2 additional years, at which time the cat was euthanized due to progressive CKD (IRIS stage 4) and an associated decline in quality of life (7). Necropsy revealed bicavitary effusion with severe CKD changes (including interstitial fibrosis and lymphoplasmacytic nephritis), multifocal, nodular, pulmonary interstitial fibrosis without inflammation (suspect idiopathic feline pulmonary fibrosis), cardiac interstitial fibrosis (concentric ventricular myocardial hypertrophy), and parathyroid hyperplasia as well as bone marrow erythroid hypoplasia (suspect secondary to renal disease). The underlying cause of the bicavitary effusion was not grossly or histologically apparent.

### Cat 2

A 13-year-old male castrated domestic longhair cat was presented for weight loss and inappetence of several months duration. The cat weighed 4.7 kg and had a body condition score of 3/9. On physical examination, the cat had a mildly distended abdomen and normal breath sounds in all lung fields. Dependent, pitting edema was detected in the cat's distal pelvic limbs and left thoracic limb.

A CBC revealed a moderate normocytic, hyperchromic, nonregenerative anemia (hematocrit 22.3%) and moderate lymphopenia (442/mm^3^; RR: 1,000–7,000). Serum biochemistry panel revealed azotemia characterized by a creatinine of 26 g/m^3^ and BUN of 540 g/m^3^ (7). Urinalysis revealed a urine specific gravity of 1.014 with proteinuria (750 g/m^3^). Urine protein to creatinine ratio was 1.2. Ultrasound revealed a large volume of anechoic effusion within the pleural space as well as scant peritoneal effusion and a small volume of retroperitoneal effusion bilaterally. The renal cortices were hyperechoic and thickened with decreased corticomedullary distinction, and multiple renal cortical cysts were detected bilaterally. A small volume of renal mineralization was present lining the renal pelves. An echocardiogram was performed. The right atrium appeared mild to moderately dilated with possible interventricular septal flattening and subjective dilation of the pulmonary artery (interpreted as a possible normal variant or associated with pulmonary hypertension). Evidence of diastolic dysfunction and hyperdynamic left ventricular systolic function were also noted. Thoracocentesis was performed bilaterally and a total of 400 cm^3^ of fluid was evacuated from the pleural space. The effusion was grossly clear with total protein 7 kg/m^3^ and total nucleated cell count 40/mm^3^; the effusion was characterized as a transudate.

Thoracic and abdominal CT scan was performed (Figures 3D–F). This revealed a large volume of fluid in the pleural space, mediastinum, and retroperitoneum. Following intravenous administration of iodinated contrast material, contrast was observed accumulating in the subcapsular space and leaking from the kidneys bilaterally into the retroperitoneal space with increasing volume over multiple sequential series. Bilateral renomegaly (4.1–4.5 cm in length) was detected, and the kidneys had irregular, rounded margins, heterogeneous contrast enhancement, multiple cortical cysts, and nephroliths. Both ureters were of normal size, and appropriate contrast filling of the ureters and urinary bladder was observed. Subcutaneous edema was present diffusely but most prominent caudally.

Given these findings, the pleural and retroperitoneal effusions were deemed likely to be of the same etiology, and given the contrast enhancement pattern with bilateral renal disease and subcapsular renal fluid, the primary differential was urine ultrafiltrate leakage from the kidneys. Underlying renal disease was considered most consistent with CKD (IRIS stage 2), though the renomegaly was atypical and a concurrent acute component was deemed possible (7). The patient was hospitalized overnight with a thoracostomy drain and oxygen support during recovery from anesthesia. The cat's PCV decreased to 19%, and a packed red blood cell transfusion was administered.

The cat underwent general anesthesia and exploratory laparotomy 1 day following CT scan. No significant peritoneal effusion was noted, though a marked volume of translucent fluid was present in the retroperitoneal space bilaterally. No ureteral, bladder, or diaphragmatic abnormalities were identified. Upon entrance into the retroperitoneal space, renal capsular defects were visible with fluid emanating from these defects into the retroperitoneal space. Approximately 80% of each renal capsule was excised *via* blunt dissection and monopolar electrocautery. The remaining portion of the renal capsules were then sutured to the retroperitoneal sublumbar musculature with 3-0 polydioxanone in a cruciate pattern to create bilateral nephropexies. The retroperitoneal space was further incised cranially and caudally to each kidney to promote drainage into the abdomen. The abdomen was closed routinely and a pleural drainage catheter and port system was placed. The cat recovered from general anesthesia and surgery without complication.

The cat was hospitalized for 15 days postoperatively. A renal panel submitted 2 days postoperatively showed progression of azotemia with creatinine 57 g/m^3^ and BUN 1,250 g/m^3^. Abdominal ultrasound performed 3 days postoperatively showed resolved perirenal fluid, static chronic kidney changes without evidence of ureteral obstruction or pyelonephritis, and progressive peritoneal effusion. Four days postoperatively, the cat's peritoneal and pleural effusion had creatinine values of 58 g/m^3^ and 59 g/m^3^, respectively, and its peripheral creatinine was 59 g/m^3^. Approximately 230–250 cm^3^ of fluid was removed from the pleural drainage catheter every 24–48 h throughout hospitalization. At the time of discharge 15 days postoperatively, the cat had a creatinine of 51 g/m^3^ and BUN of 1,180 g/m^3^.

The cat returned 1 day following discharge for humane euthanasia. Necropsy revealed transudative effusion of the thoracic, abdominal, and retroperitoneal cavities as well as severe CKD changes (including interstitial renal fibrosis and lymphoplasmacytic nephritis), severe bone marrow erythroid hypoplasia (suspect secondary to renal disease), myocardial fibrosis, and mild interstitial pulmonary fibrosis (consistent with post-inflammatory, chronic interstitial fibrosis of unknown etiology). The underlying cause of the effusion was not grossly or histologically apparent.

## Discussion

These two cases differ from previously described cases of urothorax in both mechanism and in clinical pathological analysis of the effusion. Obstruction, trauma, and urinoma were not observed. Feline cases of uroperitoneum are classically defined as having a fluid to blood creatinine ratio of 2:1 and a fluid to blood potassium ratio of 1.9:1 (8). The effusion to serum creatinine ratios for these cats were 1.04 and 1.00, which are much lower than the average ratio and also below a previously reported range for the ratio (1.09–19.8) (9). Effusion and serum potassium values were also similar for the first cat but not assessed for the second cat. Moreover, biochemical characteristics of urothorax in human patients typically include a low glucose level and high LDH level (9). For the cat in which the concentrations of glucose and LDH within the pleural effusion and serum were evaluated, the concentration of glucose was greater in the pleural effusion than in the serum, and the LDH concentration was greater in the serum than in the urine and the pleural effusion. Therefore, based on biochemical analysis, the pleural effusion in these cats is inconsistent with urine and urothorax.

However, in both cats, fluid was observed leaking from the kidneys through the renal capsules into the retroperitoneal space by both CT and intraoperative visualization. In one cat renal scintigraphy also demonstrated technetium from the urinary tract in the pleural space. The primary differential for these findings is that the fluid traversing from the kidneys into the pleural space was that of urinary ultrafiltrate (fluid following glomerular filtration of blood and prior to renal tubular reabsorption and secretion) rather than urine. This is consistent with the imaging findings because both Tc-DTPA and iodinated contrast material are filtered by glomeruli but not secreted or reabsorbed by renal tubules.

The bilateral renal capsular defects in these cats could represent historically ruptured perinephric pseudocysts. Perinephric pseudocysts have been well documented in cats, and generally the pseudocysts are readily visible in a subcapsular location on ultrasonographic imaging (10). The cause of perinephric pseudocysts in cats is unknown, but they often occur in cats with renal disease (11, 12). Perinephric pseudocyst fluid in cats generally is characterized as pure transudate or rarely modified transudate and not consistent with urine with creatinine concentrations similar to serum concentrations (11, 12).

In humans with urothorax, alleviation of the underlying cause (renal obstruction, urinoma, trauma) of the effusion is indicated for disease resolution; however, human cases of spontaneous pleural effusion secondary to urinary ultrafiltrate have not been described. Ultrasound-guided drainage of feline perinephric pseudocysts is uncommonly recommended due to recurrence, and treatment involves surgical resection of the pseudocyst capsule to permit peritoneal drainage of fluid (11, 13). One case report exists of a cat with a perinephric pseudocyst and secondary hydrothorax in which intrapseudocystic scintigraphy confirmed drainage from the pseudocyst into the pleural space, and exploratory laparotomy revealed a dissecting fistula from the pseudocyst through the caval foramen of the diaphragm (14). The cat's hydrothorax resolved following pseudocystectomy and subsequent unilateral nephrectomy (14). No fistula or diaphragmatic anomalies were observed in the cats of this report, and traditional surgical intervention to promote peritoneal drainage of fluid did not resolve the cats' recurrent pleural effusion. Nephrectomy was not a viable option due to the bilateral nature of disease.

Importantly, anatomic communication between the retroperitoneal space and mediastinum has been well established in multiple species including cats, and pneumomediastinum can result in pneumoretroperitoneum (15, 16). In the second cat in this case series, fluid could be seen dissecting and abutting the cranial mediastinal fat. In retrospect, fluid is also identified adjacent to the aorta and esophagus in both patients. The mediastinal pleura is thin and not usually detectable on CT, such that diagnosing mediastinal fluid is difficult in patients with pleural effusion. Given the combination of retroperitoneal effusion and mediastinal fluid, the marked pleural effusion implies an incomplete mediastinum with communication between the mediastinum and pleural space, which is reported in 47% of cats with pneumomediastinum (16). Thus, the suspected mechanism for translocation of fluid from the retroperitoneal to pleural space in these cats was through the mediastinum.

Furthermore, in both cats of this series, peripheral edema was documented in association with recurring episodes of pleural effusion and repeatedly improved following thoracocentesis. In the previous case report of hydrothorax secondary to a perinephric pseudocyst, the cat also developed peripheral edema after pseudocystectomy (14). The anatomical connection between the retroperitoneum and peripheral areas such as the pelvic limbs, *via* the extraperitoneal pelvis and subsequently femoral sheath, has been previously described (15). Other possible causes of peripheral edema such as hypoalbuminemia and fluid overload were ruled out in these cases, and the timing of peripheral edema relative to the accumulation of pleural effusion is suggestive of an anatomical link.

The cases of this report document the drainage of pure transudate fluid from the kidneys into the retroperitoneal space and subsequently the pleural space with an apparently intact diaphragm, no evidence of trauma or urinary tract obstruction, and the presence of renal capsular defects without persisting perinephric pseudocysts. No evidence of neoplasia such as lymphangiosarcoma was evident on imaging of the abdomen or thorax, exploratory laparotomy, or necropsy/histopathological evaluation, such that a neoplastic source of the effusion in these cats was ruled out. Pure transudates are typically caused by hypoproteinemia, which was diagnostically ruled out in the cats of this report. Hydrothorax as a sequella of perinephric pseudocysts or, as in these cases, ruptured perinephric pseudocysts, should now be added to the list of differential diagnoses in cats with pure transudate pleural effusion. As demonstrated in this case series, renal scintigraphy and contrast CT with delayed imaging are valuable modalities in evaluating for a renal source of pleural effusion. Renal scintigraphy has previously been used as a diagnostic aid in people with urothorax (whereby renal perfusion is followed by abnormal radiopharmaceutical uptake in the pleural space), and a previous case report utilized injection of sulfurcolloid-bound ^99m^Tc directly into a feline pseudocyst to demonstrate a communication (14, 17–20).

In conclusion, this case series provides evidence in support of ruptured perinephric pseudocysts as a potential cause of spontaneous and recurrent pure transudate pleural effusion in feline patients. Though the surgical correction method described in these cases decreased the rate of pleural effusion accumulation in the one cat with long-term follow up, it did not completely eliminate it, thereby supporting the need for further evaluation of this condition and effective treatment modalities.

## Data availability statement

The original contributions presented in the study are included in the article, further inquiries can be directed to the corresponding author.

## Ethics statement

Ethical review and approval was not required for the animal study and written informed consent for participation was not obtained from the owners because the study is a retrospective case series (describing the diagnosis, treatment, and outcomes of two cats with previously undescribed disease). No experimental research was performed. At initial visit at the authors' institution, approval/owner consent is obtained to include any data from that patient's medical records in publications. Therefore, no additional consent was required.

## Author contributions

All authors participated in the diagnosis, treatment, and/or management of the cats of this case series as well as data acquisition and manuscript preparation.

## Conflict of interest

The authors declare that the research was conducted in the absence of any commercial or financial relationships that could be construed as a potential conflict of interest.

## Publisher's note

All claims expressed in this article are solely those of the authors and do not necessarily represent those of their affiliated organizations, or those of the publisher, the editors and the reviewers. Any product that may be evaluated in this article, or claim that may be made by its manufacturer, is not guaranteed or endorsed by the publisher.
